# Testosterone Therapy Effects Adipose Distribution in Older Females Post Hip-Fracture: The STEP-HI Study

**DOI:** 10.1093/geroni/igaf122.4021

**Published:** 2025-12-31

**Authors:** Jacob Earp, Shangshu Zhao, Furong Xu, Richard Fortinsky, Jenna Bartley, Chia-Ling Kuo, George Kuchel

**Affiliations:** University of Connecticut, Farmington, Connecticut, United States; University of Connecticut, Farmington, Connecticut, United States; University of Rhode Island, University of Rhode Island, Rhode Island, United States; University of Connecticut, Farmington, Connecticut, United States; University of Connecticut School of Medicine, University of Connecticut Health, Connecticut, United States; University of Connecticut, Farmington, Connecticut, United States; University of Connecticut, Farmington, Connecticut, United States

## Abstract

With aging and injury females experience ectopic redistribution of appendicular adipose tissue (AAT) into the viscera, where visceral adipose tissue (VAT) becomes highly inflammatory and metabolically stressful. This adipose redistribution increases the risk of both reinjury and obesity-related illnesses. Strategies that can disrupt this unhealthy adipose redistribution after hip fracture injury (HF) are of great interest. We aimed to determine how testosterone therapy affects the amount and distribution of total adipose tissue (TAT) in older women following HF. This was a secondary analysis of the STEP-HI study, a multi-site randomized clinical trial that randomized older females recovering from HF to a 6-month resistance exercise intervention while taking either testosterone (EX+T, n = 35, age=79±9 years) or a placebo (EX+P, n = 31, age=76±7 years). Changes in TAT, AAT and VAT mass and percentage of TAT in each region (AAT% and VAT%) were measured using dual x-ray absorptiometry and compared between groups. No significant differences were observed between groups at baseline (p > 0.05). Over the intervention period, mean change between baseline and six months were not significantly different between groups for TAT (EX+P: 298±2002 g, EX+T: 419±2086 g, p = 0.827), AAT (EX+P: 52±1007 g, EX+T: 39±1078 g, p = 0.890) AAT% (EX+P: -0.42±1.40, EX+T: -0.52±1.67%, p = 0.718) and VAT (EX+P: 45±232 g, EX+T: -44±151 g, p = 0.091), while VAT% improved in the EX+T (-0.26±0.51%) compared to EX+P (+0.12±0.52%, p = 0.003). These results demonstrate that while testosterone treatment was unable to decrease overall adipose stores compared to exercise alone in older women recovering from HF, it may help prevent unhealthy adipose redistribution.

